# The underlying dimensionality of PTSD in the diagnostic and statistical manual of mental disorders: where are we going?

**DOI:** 10.3402/ejpt.v6.28074

**Published:** 2015-05-19

**Authors:** Cherie Armour

**Affiliations:** School of Psychology, University of Ulster, Coleraine, Northern Ireland, UK

**Keywords:** PTSD, CFA, DSM-IV, DSM-5

## Abstract

There has been a substantial body of literature devoted to answering one question: Which latent model of posttraumatic stress disorder (PTSD) best represents PTSD's underlying dimensionality? This research summary will, therefore, focus on the literature pertaining to PTSD's latent structure as represented in the fourth (DSM-IV, 1994) to the fifth (DSM-5, 2013) edition of the DSM. This article will begin by providing a clear rationale as to why this is a pertinent research area, then the body of literature pertaining to the DSM-IV and DSM-IV-TR will be summarised, and this will be followed by a summary of the literature pertaining to the recently published DSM-5. To conclude, there will be a discussion with recommendations for future research directions, namely that researchers must investigate the applicability of the new DSM-5 criteria and the newly created DSM-5 symptom sets to trauma survivors. In addition, researchers must continue to endeavour to identify the “correct” constellations of symptoms within symptom sets to ensure that diagnostic algorithms are appropriate and aid in the development of targeted treatment approaches and interventions. In particular, the newly proposed DSM-5 anhedonia model, externalising behaviours model, and hybrid models must be further investigated. It is also important that researchers follow up on the idea that a more parsimonious latent structure of PTSD may exist.

There has been a substantial body of literature devoted to answering one question: Which latent model of posttraumatic stress disorder (PTSD) best represents PTSD's underlying dimensionality? (cf. Yufik & Simms, 2010). A recent systematic literature review (Armour, Mullerova, & Elhai, under review) of confirmatory factor analytic (CFA) PTSD studies has highlighted 108 investigations conducted between 1994 and 2015 on exactly this topic. The systematic review focused on participants aged 12 and over who had been assessed using measures directly corresponding to the symptomatology outlined in both the fourth and fifth edition of the Diagnostic and Statistical Manual of Mental Disorders (DSM-IV; American Psychiatric Association [APA], 1994 and DSM-5; APA, 2013). This research summary will, therefore, focus on the literature pertaining to PTSD's latent structure as represented in the fourth (DSM-IV; APA, 1994) to the fifth (DSM-5; APA, 2013) edition of the DSM. This summary differs from the aforementioned systematic review in that it takes a historical perspective on the topic, whereas the systematic review focuses more on methodology and findings. This article will begin by providing a clear rationale as to why this is a pertinent research area, then the body of literature pertaining to the DSM-IV (APA, 1994) and DSM-IV-TR (APA, 2000) will be summarised, and this will be followed by a summary of the literature pertaining to the recently published DSM-5 (APA, 2013). To conclude, there will be a discussion with recommendations for future research directions.

Before proceeding, as highlighted by Elhai and Palmieri (2011), it is important to acknowledge some definitional caveats within this field of research; first, this body of literature examines the structure of DSM-IV and DSM-5 PTSD symptom measures rather than PTSD's structure *per se*; this is attributable to the direct correspondence of the items of these measures with the 17 and 20 PTSD symptoms as outlined in the DSM-IV and DSM-5, respectively. Secondly, the studies under review have largely sampled trauma-exposed participants irrespective of whether or not those participants meet the relevant diagnostic criteria. Moreover, as PTSD is a disorder emerging from exposure to a multitude of traumatic events, PTSD CFA studies have been conducted using data gleaned from a wide array of traumatised groups; some have focused on one particular traumatic event (e.g., sexual assault survivors; Ullman & Long, 2008) and others have chosen to sample individuals with extremely diverse trauma histories (e.g., nationally representative data sets; Armour, Carragher, & Elhai, 2013). As will become apparent in this article, a number of researchers have investigated whether conditions such as meeting PTSD's diagnostic criteria influence which of PTSD latent factor models are optimal (cf. Biehn, Elhai, Fine, Seligman, & Richardson, 2012).

## Pertinence

Elhai and Palmieri (2011) were first to specifically outline the pertinence of this line of enquiry. They highlighted four key points; first, they discussed how this endeavour has the ability to provide insight into the core constructs, which represent PTSD. This is particularly important given a body of literature, which has questioned PTSD's construct validity and distinctiveness as an independent psychiatric disorder (McNally, 2009; Spitzer, First, & Wakefield, 2007). These questions have largely arisen from research demonstrating PTSD's high rates of comorbidities with alternative psychopathologies such as mood and anxiety disorders. In particular, PTSD's symptom set contains items present in a number of diagnostic entities, including but not limited to items such as sleeping difficulties and concentration difficulties (Mchugh & Treisman, 2007; Rosen & Lilienfield, 2008; Spitzer et al., 2007). Some studies have suggested that certain PTSD items, specifically those known as dysphoria symptoms, are more related to depressive disorders than others (Elklit, Armour, & Shevlin, 2010), whereas some have suggested that no single PTSD item is more or less related to general distress than others (Marshall, Schell, & Miles, 2013).

Elhai and Palmieri's (2011) second point was that the resultant PTSD symptom sets have implications for diagnostic algorithms. Diagnostic algorithms dictate that individuals must endorse a particular number of items from each of the symptom sets of a given disorder. Thus, if the type and/or number of symptoms/symptom sets within a given disorder are altered, the corresponding diagnostic algorithm will also be altered. Ultimately, this may affect the prevalence of a disorder within the population; given that different people may or may not be in receipt of a diagnosis. Continuing to investigate CFA models of PTSD's latent structure will assist in the identification of the “correct” number and composition of PTSD symptom sets and, thus, aid in establishing the correct diagnostic algorithm pertaining to PTSD. Thirdly, Elhai and Palmieri (2011) discussed how information gleaned from CFA studies can enhance knowledge related to the etiology and maintenance of PTSD. Indeed, knowledge of particular symptom sets allows researchers to investigate risk factors pertaining to specific symptom sets (e.g., varying trauma experiences; Armour & Shevlin, 2010), assess whether a particular symptom set drives the longitudinal course of the disorder (e.g., hyperarousal; Schell, Marshall, & Jaycox, 2004), or whether a particular symptom set is more or less resistant to treatment (e.g., numbing; Asmundson, Stapleton, & Taylor, 2004). The fourth point emphasised that the identification and assessment of symptom sets will allow for the implementation of targeted treatments.

## DSM-IV/DSM-IV-TR^1^

The DSM-IV was published in 1994 and categorised PTSD's 17 symptoms across three distinct symptom sets: re-experiencing, emotional numbing, and hyperarousal. Four years subsequent to the publication of the DSM-IV, researchers published a paper that detailed an alternative latent model proposed to provide superior fit to PTSD data than the existing DSM-IV model. This model contained four rather than three symptom sets and was termed the emotional numbing model (King, Leskin, King, & Weathers, 1998). The symptom sets were termed: re-experiencing, numbing, avoidance, and hyperarousal; the creation of numbing and avoidance symptom sets were based on the separation of items originally belonging to the emotional numbing and avoidance symptom set of the DSM-IV model. The separation of these items was based on both theoretical and empirical evidence supporting their distinctiveness (Asmundson et al., 2004). This model was deemed preferential to the DSM-IV model (King et al., 1998) and remained uncontended until the publication of an article by Simms, Watson, and Doebbeling (2002). Simms et al. proposed a new alternative latent model termed the dysphoria model, which contained four symptom sets of re-experiencing, avoidance, dysphoria, and hyperarousal. The dysphoria symptom set comprised all of the items from the numbing symptom set of the emotional numbing model and three symptoms that were removed from the hyperarousal set; irritability, sleeping difficulties, and concentration difficulties. This new dysphoric symptom set was based on the premise that these items were not specific to the disorder but resembled symptoms of general distress. It was at this point that the interest in PTSD's latent structure from the field of traumatic stress grew monumentally. Indeed, several researchers have endeavoured to identify the model: the three-factor DSM-IV model, the four-factor numbing model, or the four-factor dysphoria model, provided the optimal representation of PTSD's latent structure (Charak, Armour, Elklit, Angmo, Elhai, & Koot, 2014; Rademaker, Minnen, Ebberink, Zuiden, Hagenaars, & Geuze, 2012). Of note, a number of studies have also attempted to identify the optimal latent structure of early posttraumatic responding (Hansen, Armour, & Elklit, 2012; Olff, Sijbrandij, Opmeer, Carlier, & Gersons, 2009).

The systematic review mentioned above (Armour et al., under review) detailed 97 studies comprising 126 samples, which examined 26 different DSM-IV PTSD models. Although many comparisons were made across a wide variety of models, the most popular were indeed the comparisons between the DSM-IV three-factor model with the emotional numbing model (*n*=75 samples) and the dysphoria model (*n*=64 samples), and directly between the emotional numbing model and the dysphoria model (*n*=105 samples). On the whole, both the four-factor models outperformed the three-factor model, and the dysphoria model outperformed the emotional numbing model (based on the quantity of samples, which chose one model as preferential to the other). These findings support those of a previous meta-analytic study assessing 40 PTSD data sets in which the dysphoria model was deemed the optimal fitting model (Yufik & Simms, 2010).

### Moderating variables

Given that studies appeared to be fluctuating between selecting either the emotional numbing model or the dysphoria model as the optimal representation of PTSD's latent structure, researchers began to question under what conditions one model may be deemed more preferential than the other. Indeed, researchers queried whether factors such as gender and age (Armour et al., 2011; Charak et al., 2014; Contractor et al., 2013; Hall, Elhai, Grubaugh, Tuerk, & Magruder, 2012; Wang et al., 2013), PTSD diagnostic status (Biehn et al., 2012), endorsement of PTSD's A2 (fear, helplessness, horror) criteria (Armour et al., 2011), PTSD measure (Palmieri, Weathers, Difede, & King, 2007; Yufik & Simms, 2010), choosing a worst trauma versus a global trauma history (Elhai, Engdahl, Palmieri, Naifeh, Schweinle, & Jacobs, 2009), having versus not having previous war zone deployment (Mansfield, Williams, Hourani, & Babeu, 2010), English versus Spanish language speakers (Marshall, 2004), Caucasian versus Hispanic ethnicity (Hoyt & Yeater, 2010), and time of PTSD assessment from trauma exposure (Krause, Kaltman, Goodman, & Dutton, 2007; Olff et al., 2009) influenced which model would be deemed preferential. Findings have been somewhat mixed. For example, Palmieri et al. (2007) reported that the numbing model provided superior fit to data from the Clinician-Administered PTSD Scale, whereas the dysphoria model provided superior fit using the self-report PTSD checklist (PCL). Armour et al. (2011) provided evidence that gender influences PTSDs latent structure, and Krause et al. (2007) provided evidence that the dysphoria model remains stable over time. In its totality, therefore, this body of evidence suggests that there are indeed conditions that may influence which of the latent structures are deemed optimal.

Armour et al. (under review) reviewed studies that directly compared emotional numbing and dysphoria models based on certain conditions to ascertain if one particular model appeared more favourable. In looking specifically at studies using military samples (*n*=27), they reported that 10 (37%) found preferential fit of the emotional numbing model, whereas 13 (48%) found the dysphoria model to be more optimal. Neither model was chosen as optimal across four (15%) studies using military samples. In civilian samples (*n*=74), the emotional numbing model provided superior fit compared with the dysphoria model in 24 (32%) samples, whereas the opposite was the case in 37 (50%) samples. Other conditions discussed in the systematic review include gender, studies focusing on university students, and studies utilising the PCL. What is important to acknowledge, however, is that when focusing on one particular difference, for example, which of the models provide superior fit more often, we must acknowledge that military samples may also differ in a multitude of ways, for example, two military samples may have been assessed using different measures, may have a very different gender, age, and ethnicity profile, and may have been assessed at different lengths of time since trauma.

### Dysphoric arousal model

A more recent conceptualisation of PTSD's dimensionality was introduced by Elhai, Biehn, Armour, Klopper, Frueh, and Palmieri (2011); these authors proposed a five-factor model termed the dysphoric arousal model. This model comprised factors of re-experiencing, avoidance, numbing, anxious arousal, and dysphoric arousal. In this model, the three items such as irritability, sleeping difficulties, and concentration difficulties were removed from the dysphoria factor to create a fifth distinct dysphoric arousal factor. The hyperarousal factor, comprising the remaining two hyperarousal items, was renamed anxious arousal. This model was introduced in late 2011, however, it quickly gathered interest from PTSD factor analytic researchers. Indeed, to date, this model has been assessed in comparison to the two four-factor models (emotional numbing and dysphoria) in 38 samples; notably, it was deemed optimal in 34 (89%) of the 38 samples (Armour et al., under review). Thus, the dysphoric arousal model has predominately been deemed preferential.

Interestingly, both the meta-analyses by Yufik and Simms (2010) and the recent systematic review of all available DSM-IV and DSM-IV-TR CFA studies highlight that the weight of evidence points to the superior performance of the dysphoria model over and above that of the emotional numbing model. Similarly, the weight of evidence based on the extant literature on which the dysphoric arousal model was based suggests that this model outperforms alternatives. A key feature of the dysphoric arousal model is the separation of the hyperarousal factor into dysphoric and anxious arousal. In moving from the DSM-IV to the DSM-5, this body of evidence was taken into consideration; however, it is interesting to note that the new DSM-5 model, as will be discussed below, most closely resembles the emotional numbing model and not the dysphoria model or the dysphoric arousal model. It has, however, been acknowledged in the field that perhaps the introduction of the dysphoric arousal model arrived too late for it to be fully considered in light of DSM-5 revisions.

## DSM-5

In May 2013, the newest, fifth edition of the DSM was published. For the first time in the history of PTSD's nosology in the DSM, PTSD's complete list of symptoms was officially divided across four rather than three symptom groups.^2^ These were re-experiencing, avoidance, negative alterations in cognitions and mood (NACM), and alterations in arousal and reactivity (AAR). Notably, a number of the symptom descriptions from the DSM-IV were retained although several were revised. Moreover, PTSD was now represented by 20 rather than 17 individual symptoms. Researchers in the field of traumatic stress quickly rose to the challenge of assessing whether the four symptom groupings were indeed the optimal way of categorising the DSM-5's 20 PTSD symptoms. Indeed, 13 articles have examined DSM-5 models across 14 samples (the total number of participants across studies; *n*=9,624; Armour et al. under review). In general, the DSM-5 model has provided adequate fit to the data across studies, although when compared with alternatives, it was deemed optimal in only 3 (21%) of the 14 samples.

Interestingly, in the studies identified in the systematic review, there were 18 different DSM-5 PTSD models assessed via CFA; these models comprised between one and seven factors (Armour et al. under review). The early approach was to assess the DSM-5 model (which most closely represented the emotional numbing model as noted above), against a DSM-5 version of the dysphoria model; this is unsurprising, given the mixed findings related to the DSM-IV models of PTSD. However, now that there are 20 rather than 17 PTSD items, the exact composition of a DSM-5 dysphoria model remains unclear; indeed, to date, there have been three versions of the DSM-5 model with regard to the placement of four items across the dysphoria and AAR symptom sets; these items are reckless or self-destructive behaviour, exaggerated startle response, difficulty concentrating, and sleeping difficulties. Similar to findings from the DSM-IV literature, neither model has been deemed conclusively preferential. In following the same vein of the DSM-IV literature, researchers have also assessed the DSM-5 and dysphoria models alongside a DSM-5 version of the dysphoric arousal model. The systematic review conducted by Armour et al. highlights five studies, which incorporated a DSM-5 dysphoric arousal model into their analyses; in four (80%) of these, the dysphoric arousal model was deemed preferential.

Shortly after the publication of the DSM-5, two independent research teams proposed alternative PTSD models each comprising six latent factors (Liu et al., 2014; Tsai et al., in press). Liu et al. (2014) assessed PTSD's latent structure using data from Chinese earthquake survivors (*N*=1,196). They assessed six competing DSM-5 models, including the DSM-5 four-factor model, a dysphoric DSM-5 model, a dysphoric arousal DSM-5 model, and a newly proposed six-factor model termed the anhedonia model. The latter model comprised factors of intrusion, avoidance, negative affect, anhedonia, dysphoric arousal, and anxious arousal. This model conceptualisation, therefore, included the separation of hyperarousal as per the dysphoric arousal model and the separation of the new NACM factor into “negative alterations in cognitions and mood” and “anhedonia.” Liu et al. stated that this was based on the premise that these factors represented positive and negative affect. Of all assessed models, the six-factor anhedonia model was deemed optimal.

Tsai et al. (in press) proposed an alternative six-factor model using data from a nationally representative sample of US veterans (*N*=1,484). This model was termed the externalising behaviours model and comprised a new factor of the same name. This factor consisted of two items: irritable or aggressive behaviour and self-destructive or reckless behaviour. The creation of this new factor was based on the premise that these items were characteristic of deficits in emotion regulation (see Cloitre, 2015; Ford, 2015 this issue for how emotion regulation is part of complex PTSD) and represented self-initiating behaviours (Friedman, 2013; Roberton, Daffern, & Bucks, 2012) and, thus, were distinct from other items. Similar to the anhedonia model discussed above, the externalising behaviours model also acknowledged and included factors separating hyperarousal into dysphoric and anxious arousal. This model was found preferential to alternatives in the full sample and in a sub-sample of those with lifetime PTSD and a sub-sample comprised of only female veterans.

Subsequent to the proposal of the two six-factor models, Armour et al. (2015) proposed a model which pulled together the features of both six-factor models into a hybrid model comprised of seven independent factors of re-experiencing, avoidance, negative affect, externalising behaviours, anhedonia, anxious arousal, and dysphoric arousal. Given this model combining the features of the two six-factor models, it was based on both theoretical and empirical evidence. The seven-factor hybrid model was deemed superior to the DSM-5 model, a DSM-5 dysphoric arousal model, an externalising behaviours model, and an anhedonia model. A number of studies currently under review have since deemed that the hybrid model demonstrates superior fit compared with the DSM-5 model and the two newly proposed six-factor models. These studies span traumatised groups including the US students, Chinese adolescents surviving earthquake, and trauma-exposed Chinese school pupils. To view the item mappings of the most recent latent DSM-5 PTSD models, see Table 1.

**Table 1:** Item mappings for the most recent DSM-5-based latent models of PTSD

Symptom	Model 1	Model 2	Model 3	Model 4
1. Intrusive thoughts	Re	Re	Re	Re
2. Nightmares	Re	Re	Re	Re
3. Flashbacks	Re	Re	Re	Re
4. Emotional cue reactivity	Re	Re	Re	Re
5. Physiological cue reactivity	Re	Re	Re	Re
6. Avoidance of thoughts	Av	Av	Av	Av
7. Avoidance of reminders	Av	Av	Av	Av
8. Trauma-related amnesia	NACM	NACM	NACM	NA
9. Negative beliefs	NACM	NACM	NACM	NA
10. Blame of self or others	NACM	NACM	NACM	NA
11. Negative trauma-related emotions	NACM	NACM	NACM	NA
12. Loss of interest	NACM	NACM	An	An
13. Detachment	NACM	NACM	An	An
14. Restricted affect	NACM	NACM	An	An
15. Irritability/anger	H	EB	DA	EB
16. Self-destructive/reckless behaviour	H	EB	DA	EB
17. Hypervigilance	H	AA	AA	AA
18. Exaggerated startle response	H	AA	AA	AA
19. Difficulty concentrating	H	DA	DA	DA
20. Sleep disturbance	H	DA	DA	DA

*Note*. Model 1=four-factor DSM-5 model; Model 2=six-factor externalising behaviours model; Model 3=six-factor anhedonia model; Model 4=seven-factor hybrid model; Re=re-experiencing; Av=avoidance; NACM=negative alterations in cognitions and mood; NA=negative affect; An=anhedonia; H=hyperarousal; DA=dysphoric arousal; AA=anxious arousal; EB=externalising behaviours.

### Towards fewer factors

Interestingly, the pattern in which the original model proposed by the DSM was superseded by two newly proposed models comprising additional factors mirrors that of the DSM-IV literature. However, in comparison with the DSM-IV pattern in which this process took from 1994 (DSM-IV) through 1998 (emotional numbing model), and 2002 (the dysphoria model) to 2011 (the dysphoric arousal model), the DSM-5 models were proposed within a short time of the publication of the DSM-5 and each other (May 2013 to present).

The majority of the extant research has, therefore, relied on testing models that increase the number of latent factors. One criticism related to the increasing number of latent factors is that, although statistically improving fit, the amount of incremental fit does not usually appear to be substantial. A separate line of enquiry has, therefore, postulated that perhaps a more parsimonious latent structure of PTSD may exist. This is based on the premise that the interfactor correlations of PTSD models are particularly high, and the knowledge that high correlations between two factors may represent a lack of discriminant validity (Brown, 2006; Kline, 2010). Indeed, in assessing the DSM-5 model, both Elhai et al. (2012) and Miller et al. (2013) have reported high interfactor correlation ranging from 0.89 to 0.94. In acknowledging the presence of high interfactor correlations, Forbes et al. (2015) used data from 570 traumatic brain injury survivors to assess four latent models; the DSM-5 model, a DSM-5 dysphoria model, a one-factor model, and a three-factor model corresponding to the structure implied by the DSM-IV diagnostic criteria. All of the assessed models, with the exception of the one-factor model, provided good fit to the data based on a number fit indices (root mean square error of approximation, confirmatory fit index, and the Tucker Lewis Index). The DSM-5 model provided superior fit compared with the three-factor model, and the DSM-5 model did not significantly differ in fit from the dysphoria model. As hypothesised by the authors, a number of factors produced high interfactor correlations (e.g., intrusion and avoidance=0.94). As a result, the authors assessed two- and three-factor models in which factors with high inter-factor correlations were combined. These new models provided a “high level of fit” and so, the authors stated, were “… viable alternatives to the three-factor or four-factor models in light of the latters’ “excessive factor intercorrelations” (p. 49). In providing a rationale over and above the high correlation for the combination of intrusive and arousal factors, the authors reported that intrusive and avoidance symptomatology although phenomenologically distinct, may indeed be two sides of the same coin. They also propose that the combination of NACM and AAR symptoms should be considered, based on no differences in fit uncovered in their study between the original models and models choosing to combine these items. The authors do, however, call for further investigation of the combination of factors and of the discriminant validity of such.

## Future directions

Undoubtedly, this field of research as it pertains to PTSD in the DSM-5 will continue along the same lines as that from the DSM-IV. Although some may consider this as repetitive, it is fundamentally important given that we must investigate the applicability of the new criteria and the newly created symptom sets to trauma survivors. Moreover, we must continue to endeavour to identify the “correct” constellations of symptoms within symptom sets to ensure that diagnostic algorithms are appropriate, and to aid in the development of targeted treatment approaches and interventions. Moreover, identifying the correct latent structure of PTSD allows researchers to assess which specific factors may account for comorbidity with alternative disorders. Indeed, further studies are needed in relation to the newly proposed latent structure of the DSM-5 criteria, in particular, the anhedonia model, the externalising behaviours model, and the hybrid model. It is also important that researchers follow up on the idea that perhaps a more parsimonious latent structure of PTSD may exist.

Ultimately, it is important that researchers attempt to replicate the findings reported in the extant literature (Armour et al., 2015; Liu et al., 2014; Tsai et al., in press). Although not discussed herein future studies should continue to evaluate the external validity of the PTSD models by assessing how each of the resultant factors relate to alternative psychopathologies and external correlates such as functional impairment (see Pietrzak et al., 2015). Furthermore, studies should specifically assess the prognostic utility of the models and assess how each of the resultant factors relates to treatment responses. Given the recent release of the National Institute of Mental Health (NIMH) Research Domain Criteria (RDoc), which calls for “new ways of classifying mental disorders based on behavioural dimensions and neurobiological measures,” it is also important that researchers assess whether or not there are important biomarkers implicated in PTSD and the relationship between these biomarkers with the distinct symptom sets (see also Schmidt, 2015, this issue). Finally, any knowledge gained must be disseminated as widely as possible to ensure that it is fed back into clinical practice.

## Conflict of interest and funding

There is no conflict of interest in the present study for any of the authors.

## Supplementary Material

The underlying dimensionality of PTSD in the diagnostic and statistical manual of mental disorders: where are we going?

The underlying dimensionality of PTSD in the diagnostic and statistical manual of mental disorders: where are we going?

The underlying dimensionality of PTSD in the diagnostic and statistical manual of mental disorders: where are we going?

The underlying dimensionality of PTSD in the diagnostic and statistical manual of mental disorders: where are we going?

The underlying dimensionality of PTSD in the diagnostic and statistical manual of mental disorders: where are we going?

The underlying dimensionality of PTSD in the diagnostic and statistical manual of mental disorders: where are we going?

## Footnotes

1 Note the 17 symptoms of the DSM-IV were consistent in the DSM-IV-TR and so will be referred to as DSM-IV symptoms.

## Footnotes

2 Note there were a number of additional changes such as changes to the definition of the traumatic stressor and the addition of a dissociative subtype; however, these will not be discussed in the present review.
