# Supplementary material for: The underlying dimensionality of PTSD in the diagnostic and statistical manual of mental disorders: where are we going?
Source: Eur J Psychotraumatol. 2015 May 19;6:10.3402/ejpt.v6.28074. doi: 10.3402/ejpt.v6.28074 (PMC4439421; doi:10.3402/ejpt.v6.28074)
Supplement: The underlying dimensionality of PTSD in the diagnostic and statistical manual of mental disorders: where are we going? [file EJPT-6-28074-s001.pdf]

La dimension sous-jacente de l'ESPT dans le manuel diagnostique et statistique des troubles mentaux : Où allons-nous ?

Cherie Armour

Une littérature abondante s'est consacrée à répondre à une question: quel modèle latent de l'état de stress post-traumatique (ESPT) représente le mieux la dimensionnalité sous-jacente de l'ESPT? Ce résumé de recherche se concentrera donc sur la littérature relative à la structure latente de l'ESPT comme représentée par la quatrième (DSM-IV; 1994) et encore présente dans la cinquième édition (DSM-5, 2013) du DSM. Cet article va commencer par fournir une justification claire pour expliquer pourquoi cette question de recherche est pertinente, puis le corps de la littérature portant sur le DSM-IV (APA, 1994) et le DSM-IV-TR (APA, 2000) sera résumé, et sera suivi par un résumé de la littérature relative à la récente publication du DSM-5 (APA, 2013). Pour conclure, il y aura une discussion avec des recommandations pour les futures directions de recherche, à savoir que les chercheurs devraient examiner l'applicabilité des nouveaux critères du DSM-5 ainsi que des nouveaux symptômes relatifs aux survivants de traumatismes créés par le DSM-5. En outre, les chercheurs devraient s'efforcer de continuer à identifier les constellations «correctes» de symptômes au sein des sets de symptômes, pour s'assurer que les algorithmes de diagnostic sont appropriés, et aident au développement d'approches et interventions thérapeutiques ciblées. En particulier, le modèle d'anhédonie nouvellement créé par le DSM-5, le modèle d'extériorisation de comportements, et des modèles hybrides doivent être plus amplement étudiés. Il est également important que les chercheurs continuent de répandre l'idée qu'une structure latente plus parcimonieuse de l'ESPT peut exister.

Mots-clés: ESPT ; CFA ; DSM-IV ; DSM-5

Citation: European Journal of Psychotraumatology 2015, **6**: 28074 - http://dx.doi.org/10.3402/ejpt.v6.28074
