# Supplementary material for: The underlying dimensionality of PTSD in the diagnostic and statistical manual of mental disorders: where are we going?
Source: Eur J Psychotraumatol. 2015 May 19;6:10.3402/ejpt.v6.28074. doi: 10.3402/ejpt.v6.28074 (PMC4439421; doi:10.3402/ejpt.v6.28074)
Supplement: The underlying dimensionality of PTSD in the diagnostic and statistical manual of mental disorders: where are we going? [file EJPT-6-28074-s002.pdf]

Die latente Dimensionalität der PTBS in der diagnostischen und statistischen Manual psychischer Störungen: Wohin gehen wir?

Cherie Armour

Mittlerweile gibt es eine substanzielle Menge an Literatur, die sich der Antwort auf die Frage: welches latentes Modell der Posttraumatischen Belastungsstörungen (PTBS) repräsentiert am besten die darunterliegenden Dimensionen? Die Forschungsübersicht wird daher auf Literatur fokusieren, die die latente Struktur der PTBS nach DSM-IV und DSM-IV-TR untersucht. Die Artikel beginnt mit einem klaren Rationale, warum diese Forschungsfrage relevant ist, und dann den DSM-IV sowie den DSM-5 bezogenen Forschungsstand summieren, gefolgt von einer Zusammmenfassung der aktuellen unlängst publizierten Artikel, die sich auf das DSM-5 beziehen. Abschließend werden Empfehlungen für zukünftigen Forschungsfragen diskutiert, insbesondere dass Forscher die Anwendbarkeit der neuen DSM-5 Kritierien und der neu entwickelten DSM-5 Symptome für Trauma-Überlebende untersuchen sollen. Weiters sollten die Forscher bestrebt sein, weiterhin nach korrekten Konstellationen von Symptomen innerhalb der Symptomssets zu suchen, um den diagnostischen Algorithmus abzusichern, un um die Entwicklung nach spezifischen Behandlungen und Interventionen zu unterstützen. Insbesondere das neu vorgeschlagene DSM-5 Anhedonie Modell, externalisierende Verhaltensmodelle, hypbrid-Modelle sollten untersucht werden. Ebenfalls wichtig erscheint es, dass die Forschung der Idee nachgehen, das eine sparsamere latente Struktur einer PTBS existiert.

Schlüsselwörter: PTBS; KFA; DSM-IV; DSM-5

Citation: European Journal of Psychotraumatology 2015, **6**: 28074 - http://dx.doi.org/10.3402/ejpt.v6.28074
