# Supplementary material for: The underlying dimensionality of PTSD in the diagnostic and statistical manual of mental disorders: where are we going?
Source: Eur J Psychotraumatol. 2015 May 19;6:10.3402/ejpt.v6.28074. doi: 10.3402/ejpt.v6.28074 (PMC4439421; doi:10.3402/ejpt.v6.28074)
Supplement: The underlying dimensionality of PTSD in the diagnostic and statistical manual of mental disorders: where are we going? [file EJPT-6-28074-s003.pdf]

A PTSD dimenzionalitása a DSM-ben: Merre tartunk?

Cherie Armour

Nagyméretű irodalom gyűlt össze ez idáig egyetlen kérdés megválaszolására: Vajon melyik modell írja le legpontosabban a poszttraumás stressz zavar látens struktúráját? Ez a kutatási összefoglaló a PTSD látens struktúrájára vonatkozó irodalom áttekintését nyújtja a DSM negyedik (DSM-IV; 1994) és ötödik (DSM-5, 2013) kiadására vonatkozóan. A cikk a téma fontosságának alátámasztásával indul, majd áttekintésre kerülnek a DSM-IV (APA, 1994) és a DSM-IV-TR-ral (APA, 2000) kapcsolatos kutatási eredmények, végül az újonnan megjelent DSM-5-el (APA, 2013) kapcsolatos vizsgálatokat mutatják be. Összefoglalásul, a diszkusszióban javaslatokat tesznek a jövőbeli kutatások témáira, mégpedig arra, hogy az új DSM-5-ös kritériumok tünetcsoportjai mennyire alkalmazhatóak trauma túlélőknél. Továbbá, a szerző kiemeli, hogy a kutatóknak feladata az, hogy igyekezzenek azonosítani a „helyes" tünetcsoportokat a minél pontosabb diagnosztikus algoritmusok és terápiás intervenciók érdekében. A DSM-5-ben leírt tünetekre vonatkozó anhedónia, externalizációs viselkedés és ezek hibrid modelljeinek kiértékelése meg kell hogy történjen. Fontos továbbá, hogy a kutatók azt a felvetést se vessék el, hogy a PTSD-nek egy ezeknél jóval egyszerűbb látens struktúrája is lehet.

Kulcsszavak: PTSD; megerősítő faktorelemzés; DSM-IV; DSM-5

Citation: European Journal of Psychotraumatology 2015, **6**: 28074 - http://dx.doi.org/10.3402/ejpt.v6.28074
