# Supplementary material for: The underlying dimensionality of PTSD in the diagnostic and statistical manual of mental disorders: where are we going?
Source: Eur J Psychotraumatol. 2015 May 19;6:10.3402/ejpt.v6.28074. doi: 10.3402/ejpt.v6.28074 (PMC4439421; doi:10.3402/ejpt.v6.28074)
Supplement: The underlying dimensionality of PTSD in the diagnostic and statistical manual of mental disorders: where are we going? [file EJPT-6-28074-s004.pdf]

# La dimensionalidad subyacente del TEPT en el manual diagnóstico y estadístico de trastornos mentales: ¿Hacia dónde vamos?

Cherie Armour

Ha habido gran cantidad de literatura dedicada a responder una pregunta: ¿Qué modelo latente de trastorno de estrés postraumático (TEPT) representa mejor la dimensionalidad subyacente del TEPT? Por tanto, este resumen de investigación se centrará en la bibliografía relacionada con la estructura latente del TEPT como se indica en la cuarta (DSM-IV; 1994) y la quinta (DSM-5, 2013) edición del DSM. Este artículo comenzará proporcionando una justificación clara de por qué es un área de investigación pertinente, después se resumirá la literatura relacionada con el DSM-IV (APA, 1994) y el DSM-IV-TR (APA, 2000), seguido de un resumen de la bibliografía relacionada con el DSM-5 (APA, 2013), publicado recientemente. Para finalizar, habrá un debate con recomendaciones sobre futuras líneas de investigación, a saber, que los investigadores deben estudiar la aplicabilidad de los nuevos criterios del DSM-5 y los nuevos grupos de síntomas de los sobrevivientes de trauma que han sido creados para el DSM-5. Por otra parte, que los investigadores deben continuar esforzándose por identificar las constelaciones "correctas" de síntomas dentro de los grupos de síntomas para garantizar que los algoritmos diagnósticos sean apropiados y ayudando en el desarrollo de intervenciones y enfoques terapéuticos específicos. En concreto, el modelo de anhedonia propuesto recientemente en el DSM-5, el modelo de conductas de externalización y los modelos híbridos deben investigarse más a fondo. También es importante que los investigadores den seguimiento a la idea de que puede existir una estructura latente más parsimoniosa en el trastorno de estrés postraumático.

Palabras clave: TEPT; CFA; DSM-IV; DSM-5

**Citation:** European Journal of Psychotraumatology 2015, **6**: 28074 - http://dx.doi.org/10.3402/ejpt.v6.28074
