# Supplementary material for: The underlying dimensionality of PTSD in the diagnostic and statistical manual of mental disorders: where are we going?
Source: Eur J Psychotraumatol. 2015 May 19;6:10.3402/ejpt.v6.28074. doi: 10.3402/ejpt.v6.28074 (PMC4439421; doi:10.3402/ejpt.v6.28074)
Supplement: The underlying dimensionality of PTSD in the diagnostic and statistical manual of mental disorders: where are we going? [file EJPT-6-28074-s005.pdf]

Ruhsal Bozuklukların tanısal ve istatistiksel el kitabında TSSB'nin altındaki boyutluluk: Nereye gidiyoruz?

Cheri Armour

Bir soruyu açıklamaya adanmış önemli bir literatür bulunmaktadır: Hangi gizil TSSB modeli TSSB'nin altında yatan boyutları en iyi temsil eder? Bu yüzden, bu araştırma özeti DSM'nin dördüncü (DSM-IV; 1994) basımından beşinci basımına (DSM-V, 2013) kadar sunulduğu gibi TSSB'nin gizil yapısı ile ilgili literatür üzerine odaklanacaktır. Bu makale bunun neden geçerli bir araştırma alanı olduğuna dair açık bir mantıklı açıklama sunarak başlayacak, sonra DSM-IV (APA, 1994) ve DSM-IV-TR (APA, 2000) ile ilgili literatür özetlenecek, bu da yakın zamanda DSM-5 (APA, 2013) ile ilgili yayınlanan literatürün özetlenmesi ile devam edilecek. Son olarak, gelecek çalışmalar için yönerge, örneğin araştırmacıların DSM-5 kriterlerinin uygulanabilirliğini araştırmaları gerektiği ve yeni oluşturulan DSM-5 kriterlerinin travma mağdurlarına ayarlanması gibi, önerilerle birlikte tartışma yer alacaktır. Bunun yanında, araştırmacılar hedefe yönelik tedavi yaklaşımları ve müdahalelerin geliştirilmesinde yardımcı olmak için ve tanısal algoritmaların uygun olduğunu kesinleştirmek amacıyla semptom gruplarının içindeki 'doğru' semptom kümelerini tanımlamak için çabalamaya devam etmeleri gerekmektedir. Özellikle, yeni sürülen DSM-5 anhedoni modelinde, dışa vurulmuş davranış modeli ve hibrid modeller ileride araştırılmalıdır. TSSB'nin daha kısa ve öz gizil yapısının olabileceği fikrinin üzerine devam etmelidirler.

Anahtar Kelimeler: TSSB; CFA; DSM-IV; DSM-5

Citation: European Journal of Psychotraumatology 2015, **6**: 28074 - http://dx.doi.org/10.3402/ejpt.v6.28074
