# Supplementary material for: The underlying dimensionality of PTSD in the diagnostic and statistical manual of mental disorders: where are we going?
Source: Eur J Psychotraumatol. 2015 May 19;6:10.3402/ejpt.v6.28074. doi: 10.3402/ejpt.v6.28074 (PMC4439421; doi:10.3402/ejpt.v6.28074)
Supplement: The underlying dimensionality of PTSD in the diagnostic and statistical manual of mental disorders: where are we going? [file EJPT-6-28074-s006.pdf]

**Osnovi dimenzionalnosti PTSP-a u dijagnostičkom i statističkom priručniku za mentalne poremećaje: kuda idemo?**

Cherie Armour

Značajan deo literature je posvećen odgovoru na jedno pitanje: koji latentni model PTSP-a najbolje odražava dimenzionalnost koja je u osnovi ovog poremećaja? Ovaj sažetak istraživanja se fokusira na literaturu koja se odnosi na strukturu PTSP-a, kako je prikazano od četvrtog (DSM-IV; 1994) do petog (DSM-5, 2013) izdanja DSM-a. Ovaj članak počinje davanjem jasnog obrazloženja zašto je ovo značajna istraživačka oblast, potom će biti sumirana literatura koja se odnosi na DSM-IV (APA, 1994) i DSM-IV-TR (APA, 2000) što će biti praćeno sumiranjem literature koja se odnosi na skoro objavljeni DSM-5 (APA, 2013). Da zaključimo, diskutovaćemo i daćemo preporuke za dalja istraživanja, a to je da istraživači moraju ispitivati primenljivost novih DSM-5 kriterijuma i novokreiranih DSM-5 setova simptoma osoba koje su preživele traumu. Čak šta više, istraživači moraju nastojati da identifikuju “ispravne” konstelacije simptoma unutar pojedinačnih setova simptoma da bi obezbedili prikladne dijagnostičke algoritme i pomogli u razvoju ciljanih terapijskih pristupa i intervencija. Posebno, novopredloženi DSM-5 model anhedonije, eksternalizovani bihejvioralni model i hibridni modeli se moraju dalje ispitivati. Takođe je značajno i da istraživači slede ideju da možda postoji šturija forma latentnog PTSP-a.

Ključne reči: PTSP; CFA; DSM-IV; DSM-5

**Citation:** European Journal of Psychotraumatology 2015, **6**: 28074 - http://dx.doi.org/10.3402/ejpt.v6.28074
